# Psychosocial Risk Factors, Burnout and Hardy Personality as Variables Associated With Mental Health in Police Officers

**DOI:** 10.3389/fpsyg.2018.01478

**Published:** 2018-09-18

**Authors:** Beatriz Talavera-Velasco, Lourdes Luceño-Moreno, Jesús Martín-García, Yolanda García-Albuerne

**Affiliations:** Department of Social, Organizational and Differential Psychology, Faculty of Psychology, Complutense University of Madrid, Madrid, Spain

**Keywords:** psychosocial risk factors, burnout, personality, police officers, regression, mental health

## Abstract

Among the variables associated with occupational stress, two of the most studied are the adverse perceptions of psychosocial risk factors in the workplace and burnout. With the rise of positive psychology, other variables of the individual type, such as hardy personality have also been the subject of study. No studies have been found that jointly examine these variables related to mental health in police officers. The aim of this research was to analyze which variables were associated with mental health in police officers. A total of 223 policemen (202 men and 21 women) participated in a cross-sectional study. Of all the variables, emotional exhaustion and perception of problems as challenges were the only factors introduced in the regression model. These factors must be considered to improve both human resource interventions and occupational health practices in this professional group.

## Introduction

Police officers are highly likely to suffer from work-related stress due to the obligations and tasks of their work, such as providing help, mediating conflicts between citizens, organizing traffic or reporting offenses. These tasks imply that the police officers are exposed to traumatic situations (Garbarino et al., 2011; Korre et al., 2014; Patil et al., 2014), and that being more likely to present with health problems as a result (van der Velden et al., 2013). Gerber et al. (2014) analyzed the work-related stress and perceived health in 460 police officers, concluding that those who felt more stressed out ended up in worse health. Lack of resources in moments of pressure, such as lack of time and human or material resources, are related to a worse perceived health (Tuckey et al., 2012). In order to explain the occurrence of work-related stress, the theoretical models most used in research have been The Demand Control Social Support model (Karasek and Theorell, 1990) and the Effort Rewards Imbalance model (Siegrist, 1998). These models state that workers experience stress, on the one hand, when he/she perceive excessive work demands and little control over them, and, on the other hand, when they note an imbalance between the efforts invested in their job and the rewards obtained in return. These models identify certain components, called psychosocial risk factors (for example, perception of: lack of control over tasks, excessive work demands, lack of organizational support, few rewards), a prolonged exposure to which is related chronic stress or burnout. The demands labor resources model (Bakker and Demerouti, 2013) has also been used in recent years to explain the occurrence of work-related stress. This model indicates that there are underlying psychological processes that play an important role in the development of work pressure and motivation. The progressive process of deterioration of health, poorly-organized jobs or chronic work demands (such as overload or emotional demands) consume the physical and psychological resources of the workers and can deplete their energy. Labor resources have a potential motivator of the intrinsic and extrinsic nature, which lead to efforts toward the achievement of objectives. On the contrary, the transactional model (Lazarus and Folkman, 1984) points out to the importance of cognitive processes and the perception of the person, in addition to individual differences as key elements in the development of stress. Thus, it could be said that the reaction to stress is highly personalized (Lecic-Tosevski et al., 2011). Researchers have pointed out to the relevance of individual differences to explain how a person perceives and deals with a stressful situation. This idea is perhaps best represented in occupational psychology by the construct known as hardy personality, which describes the predisposition to show resistance to the detrimental effects of stressors and effectively adapt to meet the demands of the environment (Kobasa et al., 1982). In general research, hardy personality has been one of the studied individual variables that has been the most-related to work stress, partly due to the rise of positive psychology within the field of Work and Organizational Psychology (Bakker et al., 2012; Moreno-Jiménez et al., 2012).

The following concepts are explained below: psychosocial risk factors, burnout, and hardy personality.

### Psychosocial Risk Factors at Work

The lack of social support within the organization is one of the variables that has best explained stress in police officers. The feeling of perceived support from peers and superiors is crucial for these professionals (Page and Jacobs, 2011). In addition, police officers are at high risk for cardiovascular disease if they perceive low control, high demands, or pressure to carry out tasks and poor support (Hartley et al., 2011; Duxbury et al., 2015). Likewise, the perceived imbalance between the efforts invested and the rewards received is related to work stress. Some studies have found that police officers who perceived low control, little organizational support, and few rewards had a greater number of symptoms associated with mental disorders such as depression, compared with those police officers who did not perceive those stressors adversely (Garbarino et al., 2013; Hoven et al., 2015). These variables (perception of excessive job demands, low control, little support, or few rewards) are psychosocial risk factors. These are defined as the organizational and psychosocial factors of organizations that can leads maladaptation, tension, or psychophysiological stress responses and are likely to adversely affect health. Examples include low participation in decision making within the organization, lack of control over work, unplanned work schedules, overwork, physical or social isolation, perceived excess job demands (or very few), or job uncertainty (Benavides et al., 2002). The first hypothesis could, therefore, be established, which is as follows:

Hypothesis 1: Psychosocial risk factors will predict psychological health in police officers.

### The Concept of Burnout

The term “burnout” refers to a situation of chronic work stress with negative affective connotations that makes it difficult for workers to perform their work and maintain their relationships that are established among them. It is a psychological syndrome whose main dimensions are emotional exhaustion, depersonalization, and feeling of lack of personal accomplishment (Maslach and Leiter, 2008). Schaufeli et al. (2009) pointed out that in this syndrome, the following characteristics prevail: (a) dysphoric symptoms, especially tiredness or emotional exhaustion (which implies negative aspects to health); (b) there is a greater number of mental and behavioral symptoms than physical symptoms (for this reason, it is a psychic phenomenon); (c) it must be related to work. The symptoms appear in relation to work situations in individuals who previously did not report psychosocial alterations; and finally, (d) there is an observable decrease in work performance due to attitudes and negative behaviors toward work. Police stress is directly related to the burnout syndrome, an association that is moderated by some personal variables such as the control locus. If the police officers present high levels of internal control locus, this situation would moderate the association between stress and burnout, in the sense that the levels of these two variables decrease, if we take into account the internal control locus (Wang et al., 2014). In Swedish police officers, the prevalence of burnout was high leading to emotional exhaustion and depersonalization, compared with other studies conducted in Norway or the Netherlands (Backteman-Erlanson et al., 2013). It is important to point out that police officers exposed to a psychosocial environment with high psychological demands, low control for decision making, poor social support from the organization, and inadequate coping strategies show high scores on emotional exhaustion and depersonalization of burnout (Padyab et al., 2016). Additionally, Smoktunowicz et al. (2015) concluded that job demands were related to high levels of burnout when social support was low.

Hypothesis 2: The three dimensions of burnout (emotional exhaustion, depersonalization, and personal accomplishment) will predict psychological health in police officers.

### Hardy Personality or “Hardiness”

Hardy personality is the set of attitudes and strategies that establishes the motivation to perceive stressful working circumstances as opportunities of development or growth. It is a variable or personal resource used to alleviate or minimize the negative effects of stressful events on health, especially to protect against occupational stress (Kobasa et al., 1982). This construct consists of three dimensions (Kobasa, 1979): commitment, control, and challenge, which are interrelated, but not redundant constructs. They are described as follows: (a) Commitment: It is the tendency to develop behaviors characterized by personal involvement in all life activities, and it is a variable proposed as a moderator of the effects of stress. In addition, this quality is not limited to a feeling of personal competence, but includes a feeling of community and/or cooperation; (b) Control: It has been the most studied dimension of the model proposed by Kobasa. It refers to the conviction that the subject has to be able to influence the course of events. This capacity of control allows individuals to perceive, in many of the stressful events, predictable consequences to their own activity and, therefore, they perceive that they can handle the stimuli to their own benefit, reducing the effects of stress. Researchers have recognized the need for people to perceive control and determined that this need is inherently beneficial (Wang et al., 2010); and, finally, (c) Challenge: It refers to the belief that change, versus stability, is the usual feature of life. From this point of view, a stressful stimulus would be understood as an opportunity or an incentive for personal development and not as a threat. The benefits of the hardy personality have been found in a wide variety of occupations, including police personnel (Barton et al., 2004). Police officers are exposed to traumatic events that are part of their organizational tasks. Traumatic exposures cause over time, the onset of stress, anxiety, and depression. However, this does not happen in all occasions. Positive results appear when police officers use their strategies or psychological skills as the hardy personality to function in stressful environments or traumatic situations (Escolas et al., 2013).

Hypothesis 3: Hardy personality factors (control, commitment, and challenge) will predict mental health in police officers.

As mentioned earlier, we have found no previous studies on the prediction of police officers that comprise psychological health that simultaneously consider predictor variables such as perception of psychosocial risk factors, burnout dimensions, and hardy personality. The earlier mentioned hypotheses can be summed up in a single one:

Hypothesis: Adverse perception of psychosocial risk factors (excessive demands, poor control, poor organizational support, and few rewards), burnout dimensions (emotional exhaustion, depersonalization, and personal accomplishment), and hardy personality factors (control, challenge, and commitment) will predict mental health.

## Materials and Methods

### Participants

In this research, a non experimental, cross-sectional design was used. Cross-sectional studies are defined at a specific point in time and their purpose is to establish associations between variables and evaluate prevalence issues, not to seek cause-and-effect relationships (Ato et al., 2013). Non probabilistic quota sampling was used in this research. Those police officers who participated voluntarily were chosen, but, as conditions, they had to present a job seniority of more than 1 year in their work position and belong to municipalities of the north, center, and south of the Community of Madrid. The total sample of the study was composed by 223 police officers. Of the total number of participants, 202 were men (90.6%) and 21 women (9.4%). The average age was 41 years (*SD* = 7.52). The average seniority in the job was 14 years (*SD* = 13.90). The average number of hours worked per week was 38 h (*SD* = 8.87).

### Measures and Instruments

#### State of Perceived Psychological Health

In order to evaluate the perceived state of health, the Spanish adaptation of the General Health Questionnaire – GHQ-28 – (Lobo et al., 1986) was used. This instrument was created with the objective of evaluating the general state of health that the person perceives with respect to: the inability to continue carrying out normal healthy functions in their daily life and the detection of the appearance of symptoms of psychic malaise in the general population that had recent symptoms (lesser than 2 weeks). There are different versions of the questionnaire. In this study, it was decided to use the GHQ-28 version in which there are four scales of 7 items each (somatic symptoms, anxiety and insomnia, social dysfunction and severe depression). The type of correction used in the present research was the Likert scale, which indicates the frequency from the lowest to the highest of experienced symptomatology. The choice of the 28-item version for this research was due to the fact that the scales can be differentiated from each other. Although there is a version with a less number of items, some other studies have used the 28-item version (Ríos et al., 2010) because its Cronbach alpha reliability is 0.90, compared to the 12-item version, whose reliability is 0.76 in the Spanish sample (Sánchez-López and Dresch, 2008).

#### Perception of Psychosocial Risk Factors in the Work Environment

The DECORE-21 (Talavera, 2016) was applied, which evaluates the workers’ perception of the following psychosocial factors: cognitive demands, control, organizational support, and rewards. The questionnaire is composed of 21 items and in addition to the scoring on each scale, the Global Risk Index (GRI) can be calculated, which takes into account the average scores in each of the four dimensions of the questionnaire and offers a global score of perception of psychosocial risk. All items are answered through a Likert scale, with the following options: “strongly disagree,” “disagree,” “undecided,” “agree,” and “strongly agree.” A high score indicates a very adverse situation from the point of view of psychosocial risks (that is, the workers perceive excessive cognitive demands, lack of control, poor support, and few rewards in their work). The range of scores for each of the scales, as well as for the global scale, is from 100 to 500. High scores or scores close to 500 indicate that the workers perceive the psychosocial risk factors in their work environment adversely. The validity of the questionnaire was determined through confirmatory factor analysis, which confirmed the structure formed by the four factors. It presents adequate psychometric properties, and, in average, it can be completed in 10 min.

#### Burnout

The Spanish adaptation of the Maslach Burnout Inventory–Human Services Survey – MBI–HSS – (Seisdedos, 1997) was used. This self-administered questionnaire consists of 22 items with seven response options on a Likert scale ranging from 0 to 6 (0 = never; 1 = few times a year or less; 2 = once a month or less; 3 = a few times a month; 4 = once a week; and 5 = few times a week; and 6 = every day). It consists of three dimensions (emotional exhaustion, depersonalization, and personal accomplishment). Burnout would be described when high scores are obtained on the first two scales (emotional exhaustion and depersonalization), and a low score on the Personal Accomplishment Scale. In all three scales, the lowest achievable direct score is 0, while the maximum is 54 for emotional exhaustion, 30 for depersonalization, and 48 for personal accomplishment. This instrument was chosen because it has demonstrated good psychometric characteristics, presenting an alpha reliability of Cronbach between 0.70 and 0.80 in the different scales (Wheeler et al., 2011). In a meta-analysis, Aguayo et al. (2011) concluded, after analyzing 51 Cronbach’s alpha coefficients, that the reliability of the MBI scales was 0.88, 0.71, and 0.78 for emotional exhaustion, depersonalization, and personal accomplishment, respectively. The validity of the questionnaire was determined by confirmatory factor analysis, showing a good adjustment for the solution of three factors (Cañadas et al., 2014).

#### Hardy Personality

The Occupational Resilience Questionnaire was used (Moreno-Jiménez et al., 2014). It consists of 15 items that evaluate three factors: control, challenge, and commitment. The questionnaire is completed using a Likert scale, whose options range from 1 to 4: 1 = “totally disagree”; 2 = “disagree”; 3 = “agree”; and 4 = “totally agree.” The higher the score in each dimension, the more is the hardy personality. The total score the in the hardy personality is obtained from the average of the three dimensions that compose the global construct, whose scores also range from 1 to 4. This questionnaire was selected since it presents a reliability between 0.74 and 0.81 and suitable validity, corroborating the three dimensions (Moreno-Jiménez et al., 2014).

### Procedure

The research exceeded all the parameters of the Deontology Commission of the Faculty of Psychology of the Complutense University of Madrid, obtaining a favorable report. The collaboration between the university and the police became effective after the signing of an agreement between both institutions. We were able to collaborate after different negotiations with the police management, since it is difficult to access this professional group to deal with work-related stress issues. The agreement emphasized on the preservation of the anonymity of the police officers and the purpose of the university to use the data for a purely scientific and research aim, without damage to the participating policemen or the organization. The battery of questionnaires was prepared, and the police officers were given an open envelope with an information sheet, informed consent, and questionnaires to be completed. All informed consents of each participant were obtained. Once the participants had completed the questionnaires and checked that all items had been rated, the researcher double-checked, and the envelope was closed, with a previous review by the participant and the researcher of the answer of all items, in order to ensure that no questions were left blank. Completion of the questionnaires lasted approximately 40 min per evaluation shift. At all times, at least three researchers were present to clarify any type of doubt regarding the items.

### Data Analysis

The data analyses were performed with the statistical package SPSS for Windows in its version 22.0. Correlations, average, and standard deviation were performed for each dimension of the different questionnaires used. In order to test the hypothesis, the hierarchical regression analysis was performed. It is a series of *k* regression analysis blocks, each with one or more variables that precede it. In each phase, the increase in *R*^2^ is observed, produced by the introduction of a new block taking into account the variables introduced in the previous sequence. This change in R^2^ acquires relevance in this procedure, since an increase in it represents what each new block brings over the previous one or can eliminate the effect of what was explained by the previous blocks (Martínez-Arias et al., 2015). Other variables such as sex and age were controlled in the analysis.

## Results

**Table 1** shows the correlations, average, and standard deviation for each dimension of the evaluated constructs (see **Table 1**).

**Table 1:** Correlations between factors, mean and standard deviation (*SD*) of each questionnaire.

Factor	1	2	3	4	5	6	7	8	9	10	11	12	13	14	15	16	17
(1) Somatic symptoms	–	0.71^**^	0.47^**^	0.44^**^	0.86^**^	0.11	0.14^*^	0.21^**^	0.10	0.20^**^	0.50^**^	0.24^**^	-0.16^*^	-0.01	-0.16^*^	-0.15^*^	-0.13^*^
(2) Anxiety and insomnia		–	0.47^**^	0.49^**^	0.88^**^	0.16^*^	0.18^**^	0.37^**^	0.17^*^	0.30^**^	0.61^**^	0.34^**^	-0.18^**^	0.08	-0.13	-0.10	-0.06
(3) Social disfunction			–	0.57^**^	0.70^**^	-0.01	0.13^*^	0.21^**^	0.15^*^	0.17^**^	0.39^**^	0.18^**^	-0.19^**^	-0.02	-0.30^**^	-0.27^**^	-0.25^**^
(4) Severe depression				–	0.73^**^	-0.17	0.10	0.16^*^	0.16^*^	0.12	0.44^**^	0.22^**^	-0.16^*^	0.02	-0.20^**^	-0.18^**^	-0.15^*^
(5) Total GHQ score					–	0.07	0.18^**^	0.30^**^	0.18^**^	0.26^**^	0.62^**^	0.32^**^	-0.21^**^	0.03	-0.22^**^	-0.20^**^	-0.16^*^
(6) Cognitive demands						–	0.24^**^	0.16^*^	0.22^**^	0.55^**^	0.20^**^	0.12	0.13	0.19^**^	0.19^**^	0.18^**^	0.23^**^
(7) Control							–	0.53^**^	0.37^**^	0.79^**^	0.33^**^	0.26^**^	-0.11	-0.07	-0.17^**^	-0.18^**^	-0.18^**^
(8) Organizational support								–	0.33^**^	0.72^**^	0.49^**^	0.34^**^	-0.25^**^	-0.05	-0.18^**^	-0.38^**^	-0.25^**^
(9) Rewards									–	0.71^**^	0.25^**^	0.17^*^	0.00	-0.03	-0.07	-0.10	-0.09
(10) GRI										–	0.45^**^	0.32^**^	-0.09	-0.00	-0.10	-0.18^**^	-0.12
(11) Emotional exhaustion											–	0.53^**^	-0.20^**^	0.03	-0.15^*^	-0.29^**^	-0.17^**^
(12) Despersonalization												–	-0.21^**^	-0.10	-0.21^**^	-0.25^**^	-0.23^**^
(13) Personal accomplishment													–	0.20^**^	0.34^**^	0.35^**^	0.37^**^
(14) Control														–	0.36^**^	0.44^**^	0.75^**^
(15) Challenge															–	0.57^**^	0.81^**^
(16) Commitment																–	0.84^**^
(17) Total hardy personality score																	–
*M*	4.29	4.01	7.14	0.92	16.34	396.86	284.87	284.98	342.99	327.42	27.47	8.92	35.65	3.07	2.87	3.06	2.99
*SD*	3.52	3.77	1.91	2.37	9.44	67.21	84.01	72.16	81.89	53.48	11.22	6.27	7.60	0.48	0.50	0.49	0.39

*^∗^p < 0.05, ^∗∗^p < 0.01.*

Regarding the correlations made, the following were noteworthy: a positive and significant association was found between the somatic symptoms scale and the emotional exhaustion dimension (*r* = 0.50, *p* < 0.01). About the anxiety and insomnia scale, positive and significant correlations were found between this scale and the organizational support factor (*r* = 0.37, *p* < 0.01) and the overall risk index of DECORE (*r* = 0.30, *p* < 0.01). The anxiety and insomnia scale also correlated positively and significantly with the emotional exhaustion dimension (*r* = 0.61, *p* < 0.01) and with the depersonalization factor (*r* = 0.34, *p* < 0.01). The severe depression scale correlated positively and significantly with the emotional exhaustion dimension (*r* = 0.44, *p* < 0.01). With respect to the overall GHQ score, we found positive and significant associations in organizational support (*r* = 0.30, *p* < 0.01). As for burnout dimensions, this overall score correlated positively and significantly with emotional exhaustion and depersonalization (*r* = 0.62, *p* < 0.01 and *r* = 0.32, *p* < 0.01). Regarding the control scale of DECORE, positive and significant correlations between control and emotional exhaustion and depersonalization scales were obtained (*r* = 0.33, *p* < 0.01 and *r* = 0.26, *p* < 0.01, respectively). The score on the organizational support scale correlated positively and significantly with scores on the emotional exhaustion (*r* = 0.49, *p* < 0.01) and depersonalization (*r* = 0.34, *p* < 0.01) scales. As for the relationship between psychosocial risk factors and hardy personality, the highest correlation occurred between organizational support and commitment, which was negative and significant (*r* = -0.38, *p* < 0.01). Besides, the GRI correlated positively and significantly with the scores obtained in emotional exhaustion and depersonalization (*r* = 0.45, *p* < 0.01 and *r* = 0.32, *p* < 0.01). Finally, we found correlations between personal accomplishment and the following factors: challenge (*r* = 0.34, *p* < 0.01), commitment (*r* = 0.35, *p* < 0.01), and global hardy personality score (*r* = 0.37, *p* < 0.01).

Regarding the average scores and standard deviations obtained from the police officers of the study, in relation to the perceived psychological health, the results showed that the highest scores (indicating a higher frequency of symptoms related to a worse perceived psychological state of health) appear in the scale of social dysfunction (*M* = 7.14, *SD* = 1.91) and in the GHQ-total dimension (*M* = 16.34, *SD* = 9.44). The direct score of the questionnaire for each subscale is 21 and for the total score 84; as a result, in general, the police officers evaluated perceived a low frequency of symptoms associated with worse mental health. Regarding the perception of psychosocial risk factors, the highest scores (and which, therefore, indicate an adverse perception of psychosocial risk) are those obtained in the cognitive demands and rewards factors (397 and 343). These scores indicate that police officers perceive excessive cognitive demands and scarce rewards. Likewise, although the scores are lower than the previous ones, they perceive scarce control and little organizational support. In relation to burnout, the police officers evaluated obtained the highest scores in the dimension emotional exhaustion. In the rest of the burnout factors, the values did not exceed the average score of the scale; only in personal accomplishment factor higher scores were obtained. Finally, with respect to the hardy personality, the total number of police officers obtained scores close to 4, the control and commitment being factors in which the police officers obtained the highest scores (3.07 and 3.06, respectively). In this questionnaire, scores close to 4 indicate higher levels of hardy personality.

The results of the prediction of perceived psychological health from psychosocial risk factors (cognitive demands, control, organizational support, and rewards); the dimensions of burnout; and the factors of the hardy personality are presented in **Table 2**.

**Table 2:** Prediction of the perceived general health state scores (GHQ-total) based on psychosocial risk factors, the dimensions of hardy personality, and burnout.

Variable	*B*	ET.B	β	*R*^2^	Δ*R*^2^
Model 1				0.09	0.09
Constant	2.43	4.28			
Support	0.04^**^	0.01	0.27		
Model 2				0.12	0.03^*^
Constant	9.28	6.36			
Support	0.03^**^	0.01	0.23		
Challenge	-3.80^*^	1.48	-0.20		
Model 3				0.39	0.27
Constant	11.86	5.50			
Challenge	-3.18^*^	1.26	-0.17		
Emotional Exhaustion	0.53	0.06	0.62		

*Sequential or hierarchical regression analysis. ^∗^p < 0.05, ^∗∗^p < 0.01.*

Considering the GHQ-total score as criterion variable, the sequential or hierarchical regression analysis showed that model 3 included the variables, challenge and emotional exhaustion, accounting for 39% of the total variance of GHQ-total. When emotional exhaustion was introduced, the organizational support of model 3, which did appear in model 2, was eliminated. There was a variation in Δ*R*^2^ of 0.27 from model 2 to model 3.

## Discussion

In this study, we examined which of the factors relating to work stress, such as perception of psychosocial risk factors, burnout, and hardy personality, predicted mental health in police officers. The descriptive data indicated that the police officers perceived excessive cognitive demands and few rewards in their work. In addition, they presented moderate values of burnout and high levels of hardy personality. Among the correlations made, it was pointed out that at a higher frequency of somatic symptoms and symptoms related to anxiety, insomnia, and depression experienced, higher levels of emotional exhaustion were found in the police officers evaluated. It is noteworthy that there was a positive and significant correlation between the perception of worse mental health and higher levels of emotional exhaustion and depersonalization. It was also interesting to know that the perception of poor support at work was significantly associated with high levels of emotional tiredness. In general, the adverse perceptions of psychosocial risk factors was associated with high levels of emotional exhaustion and depersonalization. Among the perception of the psychosocial risk factors, the results found agree that other studies that suggest that police officers feel stressed and they perceived excessive demands in their work (Gu et al., 2015). The fact that the police officers of the present research perceived these work conditions in an adverse way can be related to the tasks included in this role, since it is one of the most stressful professions (Campbell and Nobel, 2009). They are obliged to carry firearms, which represents the reason why these workers are susceptible to suffer situations of risk in which themselves or third parties are involved. Such situations generate stress and produce psychological distress (Vilardell et al., 2014).

Regarding the prediction of psychological health from the indicated variables using the hierarchical regression analysis, the results showed that only the challenge and emotional exhaustion factors explained 39% of the total variance of the variable psychological health evaluated using the GHQ-total score. The inclusion of the emotional exhaustion dimension caused the organizational support factor is not included within the model. In relation to psychosocial risk factors as health predictors, the results of this research are not in line with those other studies that suggest that lack of support from peers and supervisors is one of the specific stressors that has been most associated with stress in police officers and is considered one of the best health predictors in these professionals (Violanti et al., 2014). The perception of lack of control or excessive job demands and scarce rewards did not predict psychological health. Other studies have pointed out that lack of control over work is associated with or even predicts worker’s health (including physical health).

Specifically, in line with the psychosocial risk factors involved in the demand–control–social–support and effort–rewards Imbalance models (lack of control, excessive demands, lack of support, scarce rewards), the results found in different studies reveal that demands and control factors are related to cardiovascular disease (Bishop et al., 2003). These investigations highlight the association between the lack of control and adverse perception of health; specifically, using multiple hierarchical regression analysis, it was found that a low perceived control over tasks is related to health problems in workers, showing a reduction of psychosomatic complaints by these people when the levels of control over work increase (Schmidt and Diestel, 2011). In some longitudinal studies, it has been indicated that the perception of low perceived control, combined with the perception of high demands, has negative effects on mental health (Dalgard et al., 2009), as well as that the perception of high demands and scarce support are postulated as predictors of depression and anxiety in workers (Andrea et al., 2009).

Regarding the dimensions of the burnout construct, it was the emotional exhaustion factor that appeared as a predictor of psychological health. Considering this, it is necessary to mention that other studies have also indicated similar results in police officers (Gayman and Bradley, 2013; White et al., 2015). Regarding the dimensions of the hardy personality, it was the challenge factor that was introduced as a predictor within the regression model. Resilience is considered by most researchers as a significant construct in the study of health (Hystad et al., 2011). Likewise, hardy personality is a predictor of burnout. In countries such as Portugal, a longitudinal study conducted by Garrosa et al. (2010) with a sample of 98 nurses also confirmed that the control and challenge dimensions of the hardy personality had a specific contribution as negative predictors in the development of burnout.

As for the strengths of this research, it offers results on both of the organizational and personal variables, which have been jointly taken into account for the purpose of identifying which of these factors predicted a higher frequency of symptoms related to perception of worse mental health in police officers. On the contrary, the results presented serve to improve intervention practices for work-related stress in police officers. It has been observed in this study that the perception of problems and difficult situations as challenges or emotionally exhausting factors predict mental health; therefore, interventions for occupational stress to improve psychological health in these professionals should be addressed to those factors. The results of this research are somehow expected, since a main role has always been given to the variable – control over work – and, in this case, it does not appear as a predictor of mental health. In the same way, it is necessary to mention the different limitations of this research. In the first place, this is a cross-sectional design, which makes it impossible to obtain causal relations. On the contrary, the difficulty to access this sample, in many cases prevents longitudinal studies that provide information obtained over time (Sánchez-Teruel and Robles, 2014).

In future studies, it would be advisable to increase the size of the sample in order to perform other analyses, for example, to establish differences in work stress according to sex or occupational rank that could not be carried out in this research. Some studies have shown that there are differences in police stress according to these variables (Habersaat et al., 2015; Luceño et al., 2016). In a study of police officers from the United States and Malta, findings suggest that they would gain greater benefits if they developed skills or strategies to increase psychological capital, where police chiefs required more training to provide support to police officers and thus, reduce stress (Farr-Wharton et al., 2016). Therefore, it seems that there may be differences in possible interventions to reduce stress, depending on the occupational rank. After the completion of this empirical work, further research on work-related stress in the law enforcement professionals is recommended.

## Ethics Statement

This study was carried out in accordance with the written informed consent obtained from all participants. The protocol was approved by the Deontology Commission of the Faculty of Psychology of the Complutense University of Madrid, obtaining a favorable report.

## Author Contributions

BT-V, LL-M, and JM-G contributed to the conception and design of the study. YG-A organized the database. BT-V and LL-M performed the statistical analysis. BT-V, JM-G, and YG-A wrote the first draft of the manuscript. BT-V and LL-M revised the final version of the manuscript.

## Conflict of Interest Statement

The authors declare that the research was conducted in the absence of any commercial or financial relationships that could be construed as a potential conflict of interest.
